# Use of Doppler Ultrasound for Saphenous Vein Mapping to Obtain Grafts for Coronary Artery Bypass Grafting

**DOI:** 10.21470/1678-9741-2017-0201

**Published:** 2018

**Authors:** Fillipe Campos Lopes, Oscar Willian Bomfim Oliveira, Diego Gamarra Moreira, Magaly Arrais dos Santos, Jenny Lourdes Rivas de Oliveira, Caio Bottini Cruz, Getúlio Lubanco Filho, Paulo Chaccur, Luis Carlos Bento de Souza

**Affiliations:** 1 Instituto Dante Pazzanese de Cardiologia, São Paulo, Brazil.

**Keywords:** Cardiovascular Surgical Procedures, Coronary Disease, Myocardial Revascularization, Saphenous Vein, Ultrasonography, Surgical Wound Infection, Complications

## Abstract

**Introduction:**

The great saphenous vein is widely used as a graft in coronary artery bypass grafting surgery. Complications due to saphenous vein harvesting can be minimized when using ultrasonography mapping and marking.

**Objective:**

To analyze by clinical trial the use of vascular ultrasonography to map the saphenous vein in coronary artery bypass grafting to determine viability and dissection site.

**Methods:**

A total of 151 consecutive patients submitted to coronary artery bypass surgery with the use of the great saphenous vein as a graft were selected for this prospective study. They were divided into two groups: Group 1 - 84 patients were submitted to ultrasonographic mapping and marking of the saphenous vein; Group 2 - 67 patients had saphenous vein harvested without any previous study. Both groups were coupled with follow-up on the 1^st^, 5^th^ and 30^th^ postoperative days. Primary endpoints were need for incision of the contralateral leg and wound complications within 30 days.

**Results:**

Both legs had to be incised in 6 (8.95%) patients from Group 2 (*P*=0.0067). Wound complications occurred in 33 (23.4%) patients within 30 days, 21 (35%) from Group 2 e 12 (14.8%) from Group 1 (OR 3.095, 1.375-6.944, CI 95%, *P*=0.008). Within 30 days there were 4 (2.8%) deaths, all in Group 2 (*P*=0.036).

**Conclusion:**

The use of vascular ultrasonography for mapping of the great saphenous vein in coronary artery bypass surgery has properly identified and evaluated the saphenous vein, significantly reducing wound complications and unnecessary incisions. It would be advisable to use this noninvasive and easy to use method routinely in coronary artery bypass surgery.

**Table:** 

Abbreviations, acronyms & symbols	
BMI	= Body mass index
CABG	= Coronary artery bypass grafting
PAD	= Peripheral artery disease
STS	= Society of Thoracic Surgeons

## INTRODUCTION

Atherosclerotic coronary artery disease is prevalent in the world population and coronary artery bypass grafting (CABG) surgery is one of the most accomplished and studied procedures in the history of contemporary surgery^[1]^.

Even with the use of arterial grafts, the great saphenous vein continues to be widely used, and the graft is more common in CABG surgeries^[1]^. Although the clinical examination suggests the location of the saphenous vein, especially in the calf and distal segment of the leg, its origin in the proximal thigh segments remains unknown until surgical exploration^[2]^. In addition, methods to predict the viability of this venous graft prior to surgery are not always used, which may lead to increased morbidity for the patient. This may occur if the great saphenous vein is considered unsuitable for use in CABG surgery in the perioperative period, with the need for dissection of the saphenous vein in the other leg or for obtaining an alternative graft. Complications in the operated leg are not infrequent, such as the presence of infections with wound dehiscence, paresthesia, distal edema, lymphangitis, and erysipelas^[1-3]^. There is also the possibility of ischemic ulcers in patients with associated peripheral vascular disease.

Complications related to the surgical technique occur in up to 30% of patients undergoing saphenectomy. Data from the Society of Thoracic Surgeons (STS) of 1999^[4]^, which considered only the events observed during the hospital stay, report a rate of 4.5% of complications at the saphenectomy sites. Hematoma, seroma, wound dehiscence, wound edges necrosis or infections, increase the hospital stay and delay the rehabilitation^[5,6]^. DeLaria et al.^[3]^ demonstrated a 12-day increase in hospital stay due to complications at the saphenectomy sites. There was an average increase of $ 9,900 per patient in hospital costs.

The clinical presentation of the infection may vary from localized cellulitis to an extensive involvement of soft tissues, with drainage of secretions and areas of extensive necrosis^[7]^. Infections occur in 1% to 5% of saphenectomies and the processes of delayed healing can reach 10%^[8]^.

Technical modifications were introduced to reduce complications, such as the use of incisions in the leg initiated about 5 cm above the medial malleolus, extending proximally^[9]^. Also commonly used is saphenectomy with small staggered incisions, where interspersed incisions are made with skin islands in the saphenous vein pathway, and early recovery with this technique was reported by Tevaearai et al.^[10]^. Recently, minimally invasive techniques of endoscopic saphenectomy would be related to lower rates of infection, hematoma, seroma, and shorter hospitalization time. However, new studies are needed to investigate the patency equivalence of the endoscopically dissected graft compared to classical saphenectomy^[11]^.

The use of Doppler ultrasonography for preoperative saphenous mapping may be useful in the evaluation of saphenous vein characteristics and abnormalities, such as diameter, presence of ectasias, sclerosis, thrombosis. Ultrasound studies have demonstrated anatomical changes of the saphenous vein in more than 30% of the limbs^[12]^. With precise knowledge of the anatomy of the great saphenous vein, a direct incision can be made on the marked vein segment, avoiding large dissections, detachments and, consequently, bruising^[2]^. In addition, the study of saphenous vein characteristics may guide the choice of the leg for dissection, avoiding unnecessary incisions if the graft is inappropriate for use in myocardial revascularization surgery, reducing greater risks of surgical site infection and prolonged hospital stay with consequent cost increase.

Doppler vascular ultrasonography is a noninvasive, inexpensive and easy-to-apply method for mapping the great saphenous vein. It can be performed at the bedside, with reproducible results in evaluating the quality and viability of the venous graft, and may be useful in guiding the incision in the leg for guided dissection of the great saphenous vein. This could avoid inadvertent dissection of non-viable grafts, providing the choice of the best graft, and facilitating the procedure in order to avoid inherent complications.

Thus, the present study aims to analyze the use of vascular ultrasonography to map the saphenous vein in the preoperative period of myocardial revascularization surgery to define viability and the best site for venous graft dissection, and its impact on the reduction of unnecessary incisions and complications.

## METHODS

Patients undergoing CABG surgery with the use of a large saphenous vein were selected in a prospective study, who were followed in the period from 01/14/2016 to 11/10/2016. The study was approved on 12/15/2015, by the ethics and research committee of the Instituto Dante Pazzanese, with CAAE number: 51597615500005461.

Patients were divided into two homogeneous groups, in relation to age, presence of diabetes, smoking and peripheral artery disease (PAD) and paired for ultrasound-guided saphenectomy (Group 1) *versus* right thigh saphenectomy with staggered incisions (Group 2).

The reduction in the frequency of infection, wound dehiscence, hematoma or seroma and the need for contralateral saphenectomy due to inadequate graft were analyzed.

In this study, we selected 151 patients who would undergo myocardial revascularization with the use of the great saphenous vein. In the first 84 patients (Group 1) a high-resolution two-dimensional color Doppler ultrasonography was used for the great saphenous vein mapping with a linear transducer and conductive gel. The surgical marking of the path of the great saphenous vein to oriented incision was done with permanent ink pen.

The venous mapping was performed with the patient in orthostatism, external rotation of the leg and slight knee flexion. In the patients who could not stand up, it was used a tourniquet at the thigh root with a 1-inch Penrose drain to occlude the saphenous vein distal to the saphenofemoral junction. Initially the saphenofemoral junction was found, and the great saphenous vein was followed throughout its path, distally within the saphenous envelope. Venous diameter measurements were performed: on the proximal third of the thigh, close to the saphenous arch, medial third of the thigh, knee, middle third of the leg and distal third of the leg, anterior to the medial malleolus. Saphenous vein diameters between 2 mm and 5 mm were considered normal^[3]^. Diameters smaller than 2 mm were considered unsuitable for graft use. Diameters greater than 5 mm were considered as dilatation, and above 8 mm, graft use was contraindicated. Absence of the great saphenous vein, patency, signs of chronic occlusion, thrombosis, presence of discontinuous segments, focal dilations and precise location of the vein were also evaluated as criteria for the adequacy of the conduit for a CABG surgery.

Another 67 patients underwent CABG surgery with the use of a dissected saphenous vein with staggered incisions in the right lower limb without prior ultrasonographic examination (Group 2), with similar characteristics in relation to age, presence of diabetes, smoking and PAD.

The evaluation of operative wounds in the lower limbs was performed on the 1^st^ and 5^th^ postoperative days, with direct visualization during hospital admission by the surgeon and telephone contact on the 30^th^ postoperative day. In case of doubt, the patient was advised to return to the outpatient clinic for a new visual evaluation. The presence of hematoma, seroma, wound infection, dehiscence, necrosis and need for resuturing were evaluated.

Inclusion criteria: Patients older than 18 years, who signed informed consent form and underwent CABG surgery using the great saphenous vein.

Exclusion criteria: Prior saphenectomy, emergency surgery, combined surgeries.

Primary outcomes: Need for contralateral incision due to inadequate graft in the previously selected limb, infection, suture dehiscence, seroma or hematoma at the surgical site, necrosis, need for resuturing.

In the quantitative variables we describe the mean and standard deviation, in categorical variables by absolute and relative frequencies. To verify the difference between the groups in the quantitative variables, we used the Mann-Whitney test. In qualitative variables we used Fisher's exact test to verify the association between them.

## RESULTS

The groups were matched for age, weight, height, body mass index (BMI), sex, presence of diabetes, PAD and smoking, and there was no statistical difference between the subgroups, except for weight (Table 1).

**Table 1:** Preoperative clinical characteristics of the groups.

Characteristics	Sample n=151	Group 1 n=84	Group 2 n=67	*P* value
Male sex	103 (68.2%)	55 (65.5%)	48 (71.6%)	0.483
Diabetes	81 (53.6%)	45 (53.6%)	36 (53.7)	0.999
Smoking	51 (33.8%)	29 (34.5%)	22 (32.8%)	0.864
PAD	3 (2%)	3 (3.6%)	__	0.254
Average age (years)	62.7± 8.9	61.94±9.4	63.55±8.2	0.2
Average weight (kg)	78.4±15.5	76.38±15.9	80.79±14.7	0.037
Average height (cm)	1.65±0.09	164±0.1	166±0.08	0.51
BMI (kg/m^2^)	28.7±4.9	28.1±4.8	29.3±5.0	0.21

PAD=peripheral artery disease; BMI=body mass index

During follow-up, 1 patient in the control group died on the 2^nd^ postoperative day and 10 patients on the 30^th^ postoperative day were lost, 4 of which due to death in 30 days and 6 patients due to unavailability of contact (4 patients of Group 2 and 2 of Group 1).

The right leg was selected in 125 (82.7%) patients, 59 (88.05%) in Group 2 and 66 (78.57%) in Group 1, and the left leg in 20 (13.24%) patients, 2 (2.98%) in Group 2 and 18 (21.42%) in Group 1 (Table 2).

**Table 2:** Leg selected for intraoperative saphenous dissection.

Dissected leg	Total n=151	Group 1 n=84	Group 2 n=67	*P* value
Right	125 (82.78%)	66 (78.57%)	59 (88.05%)	0.1359
Left	20 (13.24%)	18 (21.42%)	2 (2.98%)	0.0006
Both	6 (3.87%)	__	6 (8.95%)	0.0067

The need for opening of both legs occurred in 6 (3.87%) patients in Group 2, due to inadequate or nonexistent graft in the leg initially selected by the surgeon. This outcome did not occur in Group 1 (*P*=0.0067).

**Fig. 1:**
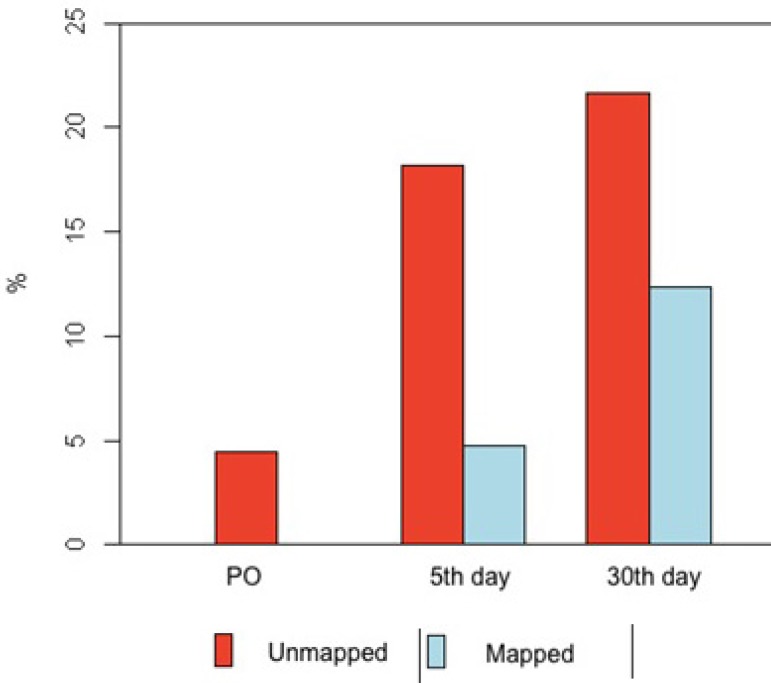
Frequency distribution bar graph of complications in groups of patients with and without mapping in the immediate postoperative period, with 5 and 30 days.

**Fig. 2:**
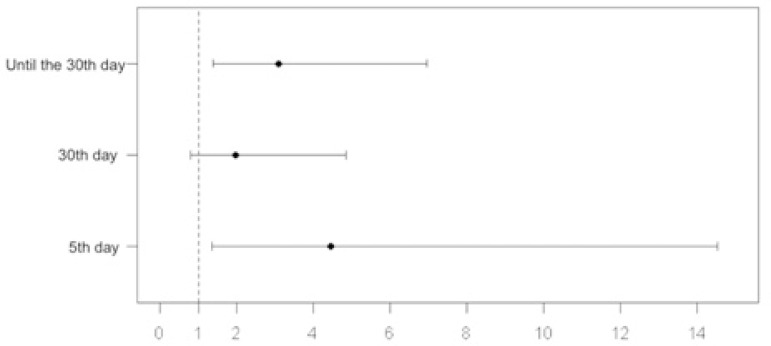
Odds ratio of patients with and without mapping on the 5^th^ and 30^th^ postoperative days and total up to the 30^th^ day.

In the follow-up of the 1^st^ postoperative period, complications in the operative wound were observed in 3 (4.5%) patients in Group 2 and no event in Group 1 (*P*=0.085). On the 5^th^ postoperative day, complications occurred in 16 (10.7%) patients, 12 (18.2%) in Group 2 and 4 (4.8%) patients in Group 1 (OR 4.444, 1.362-14.492, 95% CI, *P*=0.0014). On the 30^th^ postoperative day, complications occurred in 23 (16.3%) patients, 13 (21.7%) patients in Group 2 and 10 (12.3%) patients in Group 1 (OR 1.964, 0.796-4.854, 95% CI, *P*=0.169). In the 30-day follow-up, complications, excluding deaths, occurred in 33 (23.4%) patients, 21 (35%) in Group 2 and 12 (14.8%) patients in Group 1 (OR 3.095, 1.375-6.944, 95% CI, *P*=0.008) (Table 3, Figures 1 and 2).

**Table 3:** Complications in the operative wound of the saphenectomy.

Complications*	Total	Group 1	Group 2	*P *value
1^st^ postoperative day	3 (2%)	__	3 (4.5%)	0.085
5^th^ postoperative day	16 (10.7%)	4 (4.8%)	12 (18.2%)	0.014
30^th^ postoperative day	23 (16.3%)	10 (12.3%)	13 (21.7%)	0.169
Total in 30 days	33 (23.4%)	12 (14.8%)	21 (35%)	0.008

* Composed of hematoma, seroma, dehiscence, infection, necrosis, need for resuturing.

During follow-up, there were 4 (2.8%) deaths, all in Group 2 *(P*=0.036): a death in the 2^nd^ postoperative day due to cardiogenic shock, a death in the 5^th^ postoperative day, with cardiac arrest of unknown cause and 2 other patients during the 30-day follow-up whose exact cause was not able to be informed by the companion.

## DISCUSSION

In most cases, in both groups, the right leg was selected preferentially, following the routine of the institution. The left leg was selected in Group 2, in the presence of visible varicosity in the right leg, and in Group 1, when the saphenous vein was considered inadequate and the criteria were predetermined.

The selected groups presented similar characteristics and the difference in weight did not interfere in the BMI, which was similar in both groups. The direct impact of risk factors, diabetes, smoking, BMI, PAD on the incidence of surgical wound infection and poor healing was not assessed in this study. These factors were considered only in group correspondence.

The fact that the opening of the contralateral leg did not occur in Group 1 indicates a good accuracy of the method in identifying and evaluating the graft quality.

The follow-up stratification sought to find a higher incidence of hematoma, seroma, and suture dehiscence between the 1^st^ and 5^th^ postoperative days, which were directly related to the need for greater detachment and technical difficulties in dissection, with a significant difference of events in the 5^th^ postoperative day. On the 30^th^ postoperative day, the expectation was a higher incidence of infection, dehiscence, necrosis and need for resuturing. Despite an absolute greater number of events of these categories at this time, this was not statistically significant in the comparison between groups. This fact may be due to an insufficient sample to prove the statistical power. However, the total number of events over 30 days was significant, demonstrating benefit of the intervention.

Previous studies evaluating complications at the site of saphenectomy for CABG showed an incidence of infection similar to that found in the unmapped group. Belczak et al.^[1]^ found 25% surgical saphenectomy wound infection, where dissection was performed by staggered incisions without previous marking, whereas 31% of the complications (hematoma, seroma, infection, dehiscence) were found in the unmapped patient group. Gelape^[6]^ reported, in a literature review, similar numbers, with an incidence of complications at the site of saphenectomy reaching 30%. The complication rate of the surgical wound of saphenectomy in this study, of 12% among patients submitted to pre-marking ultrasound, reinforces the efficiency of the method in reducing events.

The deaths observed in the control group, despite having statistical significance, cannot be associated with the use of vascular ultrasonography in this study, since it was not designed with this objective. In addition, there was no consequent deaths from saphenectomy site infection.

## CONCLUSION

The use of vascular ultrasonography for the great saphenous vein mapping in isolated myocardial revascularization surgery confers an adequate accuracy in the identification of the grafts, and seems to have a good relation in the evaluation of its quality, being able to measure its dimensions with precision. In this study, we observed a lower incidence of complications related to the dissection of the great saphenous vein in the mapped patients, with statistical significance.

As a noninvasive method of easy application, it should be routinely used in myocardial revascularization procedures. New studies with cost/benefit assessment may be useful for evaluating and implementing the method in cardiac surgery services.

**Table:** 

Authors' roles & responsibilities	
FCL	Substantial contributions to the conception or design of the work; or the acquisition, analysis, or interpretation of data for the work; final approval of the version to be published
OWBO	Substantial contributions to the conception or design of the work; or the acquisition, analysis, or interpretation of data for the work; final approval of the version to be published
DGM	Substantial contributions to the conception or design of the work; or the acquisition, analysis, or interpretation of data for the work; final approval of the version to be published
MAS	Substantial contributions to the conception or design of the work; or the acquisition, analysis, or interpretation of data for the work; final approval of the version to be published
JLRO	Substantial contributions to the conception or design of the work; or the acquisition, analysis, or interpretation of data for the work; final approval of the version to be published
CBC	Substantial contributions to the conception or design of the work; or the acquisition, analysis, or interpretation of data for the work; final approval of the version to be published
GLF	Substantial contributions to the conception or design of the work; or the acquisition, analysis, or interpretation of data for the work; final approval of the version to be published
PC	Substantial contributions to the conception or design of the work; or the acquisition, analysis, or interpretation of data for the work; final approval of the version to be published
LCBS	Substantial contributions to the conception or design of the work; or the acquisition, analysis, or interpretation of data for the work; final approval of the version to be published
